# A cross-sectional study on the prevalence and determinants of antenatal anxiety among pregnant women in the second and third trimesters

**DOI:** 10.1186/s12884-026-09474-7

**Published:** 2026-06-16

**Authors:** Nisha Choudhary, Ramesh Kumar Sangwan, Mukesh Kumar, Mukti Khetan, Neha Dahiya, Bhupendra Yadav, Vikas Dhikav, Ritu Choudhary, Bontha V. Babu

**Affiliations:** 1 ICMR-National Institute for Implementation Research on Non- Communicable Diseases (NIIRNCD), Jodhpur, Rajasthan 342011 India; 2 University of Utah School of Medicine, Salt Lake City, UT USA; 3 Indian Council of Medical Research (ICMR), New Delhi, India; 4 All India Institute of Medical Sciences (AIIMS), Bilaspur, Himachal Pradesh India

**Keywords:** Psychosocial, Pregnancy, Fear of childbirth, ealthcareH, Mental health

## Abstract

**Background:**

Globally, anxiety is prevalent during pregnancy and is associated with poor outcomes, yet it is often under-documented, underdiagnosed, and untreated during antenatal care in low- and middle-income countries, and its associated factors remain poorly understood. This study aimed to estimate the prevalence of anxiety among pregnant women in their second and third trimesters and identify associated factors.

**Methods:**

A community-based cross-sectional study was conducted at four primary health centres in a rural western Indian district. Pregnant women in their second or third trimester without prior mental illness were included. Anxiety was assessed using the PRAQ-R2 tool. Data on sociodemographic characteristics, obstetric history, and various psychosocial factors were collected using the Epicollect5 application and analysed with SPSS v25.

**Results:**

Among 213 participants, 84% had pregnancy-related anxiety (60.1% mild, 22.5% moderate, and 1.4% severe). The most common PRAQ-R2 item contributing to anxiety was fear of giving birth, followed by worries about bearing an ill child, and concerns about self-appearance. Univariate analysis showed significant association with parity (crude odds ratio [COR] = 4.98, 95% confidence interval [95% CI] = 2.42-10.22), gravida (1.96, 1.04-3.71), history of abortion/intrauterine death (3.47, 1.67-7.20), negative comments or behaviour from family regarding increased appetite (1.99, 1.045-3.79), worried about specific concerns (9.42, 2.20-40.38), difficulty in reaching a healthcare facility (2.17, 1.09-4.31) and past traumatic events (1.92, 1.01-3.64), were significant. In multivariate logistic regression, parity (adjusted odds ratio, AOR = 7.83, 95% CI = 1.68-36.63), history of abortion/intrauterine death (5.08, 1.30-19.83) and worried about specific concerns during pregnancy (9.09, 1.93-42.88) remained independently associated with antenatal anxiety.

**Conclusion:**

Anxiety was highly prevalent and influenced by a complex interplay of clinical, sociodemographic, and psychosocial factors. The high prevalence in rural settings raises serious public health concerns, suggesting the need for improved screening, early identification, and timely intervention during routine antenatal care to prevent adverse pregnancy outcomes.

## Highlights

High prevalence of antenatal anxiety among pregnant women in rural India.

Fear of childbirth is the dominant component of pregnancy-related anxiety.

Nulliparity and prior pregnancy loss increase the risk of antenatal anxiety.

Psychosocial stressors strongly influence pregnancy-related anxiety.

## Introduction

Anxiety and depression are prevalent mental health disorders affecting approximately 10% of pregnant women worldwide, with a higher burden of 15-20% in low- and middle-income countries (LMICs) [1]. Specifically, anxiety is a distinct concern, with a meta-analysis reporting that 19% of women in their second trimester and 25% in their third trimester experience clinically significant symptoms [2]. Poor perinatal mental health is associated with obstetric complications, such as pre-eclampsia, haemorrhage, preterm birth, stillbirth, small-for-gestational-age, congenital malformations, protracted childbirth, and increased caesarean section rates, adversely affecting child growth and development [3, 4]. In severe cases, it may lead to suicidal ideation or suicide, a recognised cause of maternal mortality [5]. Despite these consequences, antenatal mental health disorders remain under-documented, underdiagnosed, and undertreated in LMICs, including India, where clinical attention continues to focus on obstetric complications and maternal mortality [6].

Antenatal anxiety is a distinct conditionthat is conceptually and clinically different from generalised anxiety disorder, encompassing concerns about fetal health, impending childbirth, maternal well-being during pregnancy, navigating the healthcare system, and adaptation to the maternal role [7–9]. Given these specific considerations, screening for antenatal anxiety requires validated tools such as the Pregnancy-Related Anxiety Questionnaire-Revised (PRAQ-R2), rather than general anxiety measures.

Antenatal anxiety is influenced by a complex interplay of individual, psychosocial, and environmental stressors, including psychiatric history, past traumatic experiences, lack of social or spousal support, financial hardships, and unplanned pregnancy, alongside poverty, migration, exposure to violence, and living in stressful, conflict-affected or disaster-prone environments, which may exacerbate anxiety disorders and adversely affect maternal health [4, 10, 11]. Understanding these factors within the local context is essential to developing effective strategies for early detection and supportive interventions during antenatal care (ANC).

Despite the high burden, significant consequences, and increasing global evidence, research on antenatal anxiety in India remains limited, particularly in rural settings. Most existing studies have been conducted in urban or semi-urban tertiary care facilities, whereas rural and urban populations in India differ in sociodemographic contexts, access to healthcare services and education, financial conditions, social support systems, and gender roles. To address this gap, the present study was conducted in a predominantly rural district of Rajasthan, a western Indian State, to determine the prevalence of antenatal anxiety among women in their second and third trimesters and to identify associated sociodemographic and psychosocial factors. This is one of the first community-based studies on pregnancy-specific anxiety, generating context-sensitive evidence to inform targeted antenatal mental health policies and interventions for underserved rural areas.

## Methods

### Study setting and participants

This cross-sectional study was conducted in four Primary Health Centres (PHCs) of Nagaur district, Rajasthan, between 6 January and 5 June 2025. First, a list of all PHCs in the district was obtained and four PHCs were selected using simple random sampling. Subsequently, lists of eligible pregnant women in the second and third trimesters were prepared from ANC registers and line-lists maintained by ANMs and ASHAs within the selected PHCs. Eligible women were assigned unique identification numbers, and participants were selected through simple random sampling until the required sample size was achieved. Eligible participants were pregnant women, residing in Nagaur district, who were in their second (gestational age 13-27 weeks) or third trimester (gestational age ≥ 28 weeks), identified using ANC registers at PHCs and line-lists prepared by Auxiliary Nurse Midwives (ANMs) and Accredited Social Health Activists (ASHAs). The research team and ASHAs conducted household visits to make initial contact, provide study information, and obtain informed consent. The study excluded those with a prior mental health disorder or who could not be contacted even after two household visits.

The sample size was calculated using a reported anxiety prevalence of 27% from a Mysore, Karnataka study [12], with a 5% permissible error and a 90% confidence interval (CI), was adopted to address the challenges of recruiting pregnant women in rural settings and to minimise participant burden, reflecting a balance between feasibility and statistical rigour. It yielded a minimum sample size of 213 using the equation, $n=\frac{{z}^{2}pq}{{d}^{2}}$; where, *n* is the desired sample size, *z* is the desired confidence level, *p* is the estimated proportion of anxiety in the population, *q* is *(1-p)*, and *d* is the permissible margin of error.

### Data collection

Data was collected using the Epicollect5 application, which captured sociodemographic, obstetric, antenatal health information, and PRAQ-R2 measures. A structured questionnaire, developed through a relevant literature review and expert consultation, included items on sociodemographic characteristics, obstetric history, and antenatal health-related information. Prior to data collection, a brief situation analysis was undertaken through consultations with PHC medical officers, ANMs, ASHAs, and community representatives. The assessment focused on local sociocultural norms, healthcare-seeking practices, language preferences, and logistical considerations related to accessing pregnant women in rural communities. Findings from this process informed questionnaire adaptation, field procedures, and participant engagement strategies to ensure cultural appropriateness and feasibility. The questionnaire was translated into Hindi and pilot-tested before use. When participants identified with severe anxiety, the ANM, a core provider within India’s primary healthcare system, was notified to manage the case in accordance with established ANC protocols.

Anxiety was assessed using the validated tool PRAQ-R2, a 10-item instrument developed by Huizinket al.from the original 11-item PRAQ-R [13]. The PRAQ-R2 is widely used to assess and identify pregnancy-specific anxiety across all pregnant women, irrespective of parity. It consists of three subscales: (i) *F1:Fear of giving birth* (sum of three items), e.g., ‘I am worried about the pain of contractions and the pain during delivery’, (ii) *F2: Worries about bearing a physically or mentally handicapped child* (sum of four items), e.g., ‘I sometimes think that our child will be in poor health or will be prone to illnesses’, (iii) *F3:Concern about own appearance* (sum of three items), e.g., ‘I am worried about my enormous weight gain’. Responses are rated on a 5-point Likert scale, from 1 (Absolutely not relevant) to 5 (Very relevant).

### Statistical analysis

Analyses were conducted using IBM SPSS Statistics v25. Baseline sociodemographic, obstetric, and antenatal health characteristics were summarised using descriptive statistics. For the PRAQ-R2, mean and standard deviation (SD) were calculated for each subscale item. Anxiety levels were categorised into three levels based on quartile-based classification of PRAQ-R2 scores: mild anxiety (25th to < 50th quartile), moderate anxiety (50th to < 75th quartile), and severe anxiety (≥ 75th quartile) [14]. A logistic regression analysis was performed to identify the factors associated with antenatal anxiety, categorised into no-to-mild anxiety and moderate-to-severe antenatal anxiety. Crude odds ratios (COR) and adjusted odds ratios (AOR) with 95% CIs were calculated and estimated for the variables. The statistical significance threshold was set at *p* < 0.05.

## Results

Table 1 summarises the sociodemographic characteristics of the participants. A total of 213 participants were recruited, evenly distributed by age: 49.8% aged ≤ 24 years and 50.2% aged ≥ 25 years. Approximately 22.1% of women had no formal education, whereas husbands generally had higher educational attainment. Community category was recorded according to the Government of India social classification system. “Unreserved (General)” refers to communities not eligible for affirmative action policies, whereas “Reserved” includes Scheduled Castes (SC), Scheduled Tribes (ST), and Other Backward Classes (OBC), which are recognized as socially and educationally disadvantaged groups. Most families (93%) had an annual income below ₹300,000 (~ USD2900). Occupational status was categorized as homemaker (housewife) or engaged in paid employment. The majority of women were unemployed (89.7%), while husbands were most often employed (94.4%). Extended family living was reported by 84%. Household size was ≥ 6 members in 45% of cases, and 60.1% of families lived in homes with ≤ 3 rooms. Housing type was categorized according to standard Indian census definitions. A kuccha house is constructed predominantly from temporary materials such as mud, thatch, bamboo, or unburnt bricks. A semi*-*pucca house contains both temporary and permanent construction materials. A pucca house is constructed entirely from durable materials such as cement, concrete, stone, or burnt bricks. Government hospitals were the preferred source of care for 66.2% participants, and 62.0% resided within 10 km of the nearest PHC.

**Table 1:** Distribution of sociodemographic characteristics

Variables	Category	Frequency
Age of women	≤ 24 years	106 (49.8%)
	≥ 25 years	107 (50.2%)
Age at the time of marriage (in years)	≤ 17 years	40 (18.8)
	≥ 18 years	173 (81.2%)
Education level of the respondent	No formal education	47 (22.07%)
	Formal education	166 (77.93%)
Education level of the husband	No formal education	20 (9.39%)
	Formal education	193 (90.61%)
Occupation of the respondent	Homemaker	191 (89.67%)
	Employed	22 (10.33%)
Occupation of the Husband	Employed	201 (94.37%)
	Unemployed	12 (5.63)
Religion	Hindu	188 (88.3%)
	Muslim	25 (11.7%)
Community	Unreserved (General)	23 (10.8%)
	Reserved (OBC/ST/SC)	190 (89.2%)
Annual family income (INR)	< 300,000	198 (93%)
	> 300,000	15 (7%)
Total Family members	≤ 6 members	118 (55.4%)
	> 6 members	95 (44.6%)
Type of house	Kuccha/Semi-pucca	161 (78.26%)
	Pucca	52 (24.4%)
Number of rooms	≤ 3 rooms	128 (60.1%)
	> 3 rooms	85 (39.9%)
Type of family	Extended	179 (84%)
	Nuclear	34 (16%)
Tobacco consumption among family	Yes	87 (40.8%)
	No	126 (59.2%)
Alcohol consumption among family	Yes	29 (13.6%)
	No	184 (86.4%)
Hospital visit when needed	Government hospital	141 (66.2%)
	Private hospital	72 (33.8%)
Distance to nearest health facility (PHC) (in km)	≤ 10 km	132 (62%)
	> 10 km	81 (38%)

*Abbreviations*: *INR* Indian Rupees, *OBC* Other Backward Classes, *ST* Scheduled Tribes, *SC* Scheduled Castes, *PHC* Primary Health Centre, *km* kilometer

Table 2 presents the obstetric and antenatal health characteristics of participants. In responses, 7.0% reported a history of chronic disease, and 8.9% were on medication. More than half (58.7%) were in the second trimester, and 41.3% were in the third trimester. Gravidity indicated 40.4% primigravida and 59.6% multigravida. Parity showed 48.4% nulliparous and 51.6% parous. Despite most pregnancies (73.2%) being planned, current pregnancy complications were reported by 59.6% participants, while 10.3% reported complications in previous pregnancies, and 18.8% had experienced past abortion or intrauterine death. Psychosocial stressors included insufficient family support in 45.1%, intimate partner violence (IPV) in 10.3%, family pressure to have a child in 73.7%, pregnancy-specific worries in 78%, pressure to perform household work in 72.8%, negative comments regarding increased appetite in 34.7%, and discomfort with living conditions in 62% of participants. Barriers to healthcare access during pregnancy were frequent: 59.2% reported difficulty reaching a facility, 38.5% reported family restriction on seeking care, and 66.7% reported challenges accessing or communicating with providers.

**Table 2:** Distribution of obstetric and antenatal health characteristics

Variables	Category	Frequency
History of chronic disease	Yes	15 (7%)
	No	198 (93%)
Current medication for chronic disease	Yes	19 (8.9%)
	No	194 (91.1%)
Gestational age (in weeks)	13-26	125 (58.7%)
	27-40	88 (41.3%)
Gravida	Primigravida	86 (40.4%)
	Multigravida	127 (59.6%)
Parity	Nulliparous	103 (48.4%)
	Parous	110 (51.6%)
ANC check-up at health facility	Yes	196 (92%)
	No	17 (8%)
Home visit by ASHA/ANM	Yes	29 (13.6%)
	No	184 (86.4%)
Current pregnancy planned	Yes	156 (73.2%)
	No	57 (26.8%)
Current pregnancy complications	Yes	127 (59.6%)
	No	86 (40.4%)
Previous pregnancy complications	Yes	22 (10.33%)
	No	191 (89.67%)
History of abortion/intrauterine death	Yes	40 (18.8%)
	No	173 (81.2%)
Family support during pregnancy	Yes	117 (54.9%)
	No	96 (45.1%)
Rest during pregnancy	Yes	179 (84%)
	No	34 (16%)
IPV/Domestic violence	Yes	22 (10.3%)
	No	191 (89.7%)
Past traumatic events (loss of a loved one, natural disaster, illness, injury, financial hardship)	Yes	79 (37.1%)
	No	134 (62.9%)
Antenatal anxiety due to migration	Yes	28 (13.1%)
	No	185 (86.9%)
Negative comments or behaviour from family regarding increased appetite during pregnancy	Yes	74 (34.74%)
	No	139 (65.26%)
Family pressure to perform household activities during pregnancy	Yes	155 (72.77)
	No	58 (27.23%)
Family pressure to have a child	Yes	157 (73.7%)
	No	56 (26.3%)
Pregnancy-specific worries	Yes	166 (77.93)
	No	47 (22.07%)
Family restrictions on healthcare access during pregnancy	Yes	82 (38.5%)
	No	131 (61.5%)
Difficulty in reaching a healthcare facility during pregnancy	Yes	126 (59.2%)
	No	87 (40.8%)
Challenges in accessing healthcare services with healthcare providers during pregnancy	Yes	142 (66.67%)
	No	71 (33.33%)
Discomfort with living conditions(living space, ventilation or basic facilities) during pregnancy	Yes	132 (62%)
	No	81 (38%)

*Abbreviations*: *ANC* Antenatal Care, *ASHAs* Accredited Social Health Activists, *ANMs* Auxiliary Nurse Midwives, *IPV* Intimate Partner Violence

### Mean scores of PRAQ-R2 items

Table 3 presents the mean score and SD for each PRAQ-R2 item. Among the subscales, *Fear of giving birth* (F1) had the highest mean score (mean ± SD, 10.16 ± 2.12), followed by *Worries about bearing a handicapped child* (F2; 7.88 ± 3.89), and *Concern about own appearance* (F3; 7.1 ± 4.05). The mean total PRAQ-R2 score (F1+ F2+F3) was 25.14 ± 10.06, indicating a moderate level of pregnancy-specific anxiety.

**Table 3:** Mean and standard deviationfor the PRAQ-R2 items

Subscale	PRAQ-R2 items	Item Score (Mean ± SD)	Subscale Score (Mean ± SD)	Total Score (Mean ± SD)
Fear of giving birth (F1)	I am anxious about the delivery.	3.65 ± 1.20	10.16 ± 2.12 (F1)	25.14 ± 10.06 (F1 + F2+F3)
	I am worried about the pain of contractions and the pain during delivery.	3.40 ± 1.10		
	I am worried about not being able to control myself during labour and fear that I will scream.	3.11 ± 0.94		
Worries about bearing a handicapped child (F2)	I sometimes think that our child will be in poor health or will be prone to illnesses.	1.98 ± 0.8	7.88 ± 3.89 (F2)	
	I am afraid the baby will be mentally handicapped or will suffer from brain damage.	1.86 ± 0.9		
	I am afraid our baby will be stillborn, or will die during or immediately after delivery.	1.81 ± 1.01		
	I am afraid that our baby will suffer from a physical defect or worry that something will be physically wrong with the baby.	2.23 ± 1.18		
Concern about own appearance (F3)	I am worried about the fact that I shall not regain my figure after delivery.	3.6 ± 1.22	7.1 ± 4.05 (F3)	
	I am concerned about your unattractive appearance.	2.2 ± 1.05		
	I am worried about my enormous weight gain.	1.3 ± 0.72		

*Abbreviations*: *PRAQ-R2* Pregnancy Related Anxiety Questionnaire - Revised 2, *SD* Standard Deviation, *F1* Fear of giving birth subscale, *F2* Worries about bearing a handicapped child subscale, *F3* Concern about own appearance subscale

### Prevalence of antenatal anxiety

Pregnancy-specific anxiety was prevalent among 84% of participants, with the majority experiencing mild anxiety at 60.1%, followed by moderate at 22.5% and severe at 1.4%.

### Associated factors of antenatal anxiety

Logistic regression was performed to identify associated factors of pregnancy-related anxiety. Anxiety levels were categorised into two groups: women with anxiety and without anxiety. The combined moderate-to-severe category accounted for 23.9%, and the no-to-mild anxiety category accounted for 76.1% of the sample. Based on univariate analysis, parity (COR = 4.98, 95% CI = 2.42-10.22), gravida (COR = 1.96, 95% CI = 1.04-3.71), history of abortion/intrauterine death (COR = 3.47, 95% CI = 1.67-7.199), negative family comments regarding increased appetite (COR = 1.99, 95% CI = 1.05-3.79), pregnancy-specific worries (COR = 9.42, 95% CI = 2.20-40.38), difficulty in reaching a healthcare facility (COR = 2.17, 95% CI = 1.09-4.31), and past traumatic events (COR = 1.92, 95% CI = 1.01-3.64) showed significant association with moderate-to-severe anxiety, which were further analysed using a multivariate logistic regression model. In the multivariate analysis, parity (AOR = 7.83, 95% CI = 1.68-36.63), history of abortion/intrauterine death (AOR = 5.077, 95% CI = 1.30-19.83), and pregnancy-specific worries (AOR = 9.09, 95% CI = 1.93-42.88) remained independently associated with antenatal anxiety. The Hosmer-Lemeshow goodness-of-fit test was used to assess calibration of the predictive model (*χ*^2^ = 7.359, *P* = 0.498). Table 4 presents the results of the logistic regression analysis.

**Table 4:** Logistic regression analysis of factors associated with anxiety

Variable	Category	COR (95%CI)	*p*-value	AOR (95%CI)	*p*-value
Parity	Nulliparous	4.977(2.423–10.22)	< 0.001*	7.835(1.676–36.634)	0.009*
	Parous				
Gravida	Primigravida	1.964(1.040–3.711)	0.038	0.818(0.135–4.956)	0.827
	Multigravida				
History of abortion/intrauterine death	Yes	3.471(1.674–7.198)	< 0.001*	5.076(1.299–19.833)	0.019*
	No				
Negative comments or behaviour from family regarding increased appetite during pregnancy	Yes	1.991(1.047–3.787)	0.036	2.342(0.948–5.790)	0.065
	No				
Pregnancy-specific worries	Yes	9.423(2.199–40.377)	0.003*	9.091(1.927–42.884)	0.005*
	No				
Past traumatic events	Yes	1.923(1.015–3.643)	0.045	0.752(0.308–1.836)	0.531
	No				
Difficulty in reaching a healthcare facility during pregnancy	Yes	2.168(1.089–4.315)	0.028	1.174(0.492–2.806)	0.718
	No				

*Abbreviations*: *COR* Crude Odds Ratio, *CI* Confidence Interval, *AOR* Adjusted Odds Ratio

*p < 0.05 indicates statistical significance

## Discussion

In this community-based cross-sectional study, the prevalence of pregnancy-related anxiety among antenatal women was 84% (60.1% mild, 22.5% moderate, and 1.4% severe), which is substantially higher than rates reported in previous studies in India, including 27% in Mysore, 55.7% in Bangalore and 23% tertiary South India study [12, 15, 16]. Studies from other countries reported 26.6% in Qatar, 25% in Tanzania and 32.7% in Ethiopia [17–19]. Further, a systematic review across India found antenatal anxiety prevalence ranging from 13% to 55% and a global meta-analysis reported pooled prevalence rates of 18.2% in the first trimester, 19.1% in the second trimester, and 24.6% in the third trimester, with clinically diagnosed anxiety disorders at 15.2% [2, 20]. The high prevalence identified in our study may be attributable to its rural setting. Most previous studies were conducted in urban settings, where pregnant women generally have better access to healthcare services, higher levels of education, nuclear families, and financial or occupational independence. These factors often reduce psychological stress and anxiety. In contrast, rural pregnant women such as those included in this study often face limited healthcare resources, lower educational attainment, and fewer employment opportunities, circumstances that can increase their vulnerability to pregnancy-related anxiety.

Analysis of subscale scores in this study showed higher scores for fear of giving birth compared to the Southern Pune cohort but lower scores for concerns about fetal health and apprehension about physical appearance [21]. Pregnancy-related anxiety manifests as worries about labour and childbirth, concerns regarding fetal health, and apprehension about physical appearance, with overall anxiety levels associated with these PRAQ-R2 subdomains [13]. Fear often intensifies as childbirth approaches, despite being expected throughout pregnancy. Pregnancy and childbirth represent critical transitional periods characterised by profound physiological and psychological changes. These transitions may trigger fear and vulnerability, irrespective of a woman’s prior childbirth experience [22]. Fear of childbirth is influenced by biological, social, and psychological factors. Biological contributors include parity, gestational age, pain tolerance, and high-risk maternal conditions, while social factors involve limited family support, financial hardship, and lack of partner support. Furthermore, negative obstetric experiences such as protracted labour, forceps delivery, perineal injury, abuse during childbirth, or unfavourable delivery environments can exacerbate anxiety [23].

Psychosocial stressors such as pregnancy-related worries were strongly associated with pregnancy-related anxiety in the current study. This finding is consistent with the studies conducted in Tanzania and Ethiopia [18, 19]. Perceived stress refers to an individual’s assessment of the level of discomfort caused by stressors and their ability to manage them. Expectant mothers experience stress, particularly as they near the time of delivery. This increased stress may consequently raise anxiety levels [24]. Stressors such as concerns about childbirth, financial insecurity, relationship difficulties, housing or job instability, domestic violence, family disruption, and limited social support can overwhelm a woman’s emotional coping abilities and create a cycle in which poor emotional regulation exacerbates stress, which further impairs emotional control [25]. In addition, pregnancy-specific distress refers to worries directly related to pregnancy, such as findings from prenatal screenings, nutrition and diet, baby’s growth and development, intrapartum and postpartum care, and concerns regarding adaptation to maternal roles and responsibilities [26].

Nulliparity was independently associated with pregnancy-related anxiety in the current study, which is consistent with findings from studies in the Netherlands and Iran [27, 28]. In contrast, the Bangalore study reported no significant association with parity [15], and a South African cohort identified multigravida as a significant associated factor, indicating that prior negative birth experiences may contribute to anxiety in multiparous women [29, 30]. Women who had not previously experienced childbirth often exhibit uncertainty about childbirth and worry caused by lack of familiarity with the labour process, fear and concern about pregnancy, labour pain anddelivery, health of the baby, responsibilities of future parenting, birth-related complications, and medical procedures [8, 30–33].

A history of abortion or intrauterine death was also independently associated with antenatal anxiety, consistent with findings from South Africa, Qatar and Norwegian cohorts, whereas the Bangalore study found no association for obstetric history [15, 17, 29, 34]. These experiences are often accompanied by depression, grief, guilt, sadness, excessive worry, fear of recurrence, social blame, cultural stigma, and hypervigilance regarding fetal health, thereby increasing the risk of anxiety during pregnancy [35–37].

The study has strengths, including the community-based sampling of pregnant women in their second and third trimesters and the use of the PRAQ-R2, which ensured measurement of pregnancy-specific anxiety with established psychometric validity. However, it has a few limitations, including sampling women from four PHC areas, which limits generalizability to a broader population, and the cross-sectional nature of the data, which can reveal associations between pregnancy-related anxiety and other variables mentioned earlier but cannot establish a cause-and-effect relationship. Regression analysis was conducted with covariates selected a priori based on socio-economic and clinical relevance; however, the exploratory nature of the study and the relatively modest sample size may increase the risk of model overfitting, particularly given that the aim was not to develop a prediction model or risk score. Recognising the challenges of recruiting hard-to-reach, pregnant women in rural settings and the ethical imperative to minimise participant burden, this study adopted a 90% confidence level for sample size estimation. This approach reflects a pragmatic balance between statistical rigor and feasibility, ensuring that the study remains adequately informative while avoiding unnecessary over-enrolment of a vulnerable population.

To address these findings, routine anxiety screening should be integrated into all antenatal visits at primary health care facilities [38]. Health workers’ outreach must be strengthened through more frequent home visits, particularly for high-risk women. Focused support should be provided to nulliparous women and those with prior pregnancy losses. Brief antenatal workshops on childbirth preparation, newborn care, and stress management should be offered. Timely counselling and mental health referrals are essential for women with adverse obstetric histories [39]. Additionally, research on local coping strategies among women with past trauma should be conducted to inform culturally appropriate, community-based interventions. This study may be scaled up and supplemented with targeted interventions to enhance maternal and child health outcomes.

## Conclusion

This study identified an exceptionally high rate of pregnancy-related anxiety (84%) among second and third-trimester women in Nagaur. Factors such as obstetric history, psychosocial stressors, barriers to healthcare access showed an association with anxiety. These results emphasize the urgent need for early identification and management of antenatal anxiety through routine screening, targeted counselling, and strengthened community health interventions. Addressing these factors can help alleviate pregnancy-related anxiety and minimize its harmful effects on both maternal and child health.

## Data Availability

All data generated or analysed during this study are included in this article.

## Abbreviations

ANC: Antenatal Care
ANMs: Auxiliary Nurse Midwives
AOR: Adjusted Odds Ratio
ASHAs: Accredited Social Health Activists
CI: Confidence Interval
COR: Crude Odds Ratio
F1: Fear of giving birth subscale
F2: Worries about bearing a handicapped child subscale
F3: Concern about own appearance subscale
INR: Indian Rupees
IPV: Intimate Partner Violence
KM: Kilometer
LMICs: Low- and Middle-Income Countries
OBC: Other Backward Classes
PHC: Primary Health Centre
PRAQ-R2: Pregnancy Related Anxiety Questionnaire - Revised 2
SC: Scheduled Castes
SD: Standard Deviation
ST: Scheduled Tribes
USD: United States Dollar
^*^*p* < 0.05: Indicates statistical significance

## References

[1] World Health Organization. Promoting mental health: concepts, emerging evidence, practice. Geneva: WHO; 2005.

[2] Dennis CL, Falah-Hassani K, Shiri R. Prevalence of antenatal and postnatal anxiety: systematic review and meta-analysis. Br J Psychiatry. 2017;210(5):315–23. 10.1192/bjp.bp.116.187179. 28302701 10.1192/bjp.bp.116.187179

[3] World Health Organization. WHO guide for integration of perinatal mental health in maternal and child health services. Geneva: WHO; 2022. https://www.who.int/publications/i/item/9789240057142.

[4] World Health Organization. Maternal mental health [Internet]. Geneva: WHO; 2024. https://www.who.int/teams/mental-health-and-substance-use/promotion-prevention/maternal-mental-health.

[5] Gimbel LA, Weingarten SJ, Smid MC, Hoffman MC. Maternal mental health as a major contributor to maternal mortality. Semin Perinatol. 2024;48(6):151943. 10.1016/j.semperi.2024.151943. 39095259 10.1016/j.semperi.2024.151943

[6] Rahman A, Patel V, Maselko J, Kirkwood B. The neglected ‘m’ in MCH programmes–why mental health of mothers is important for child nutrition. Trop Med Int Health. 2008;13(4):579–83. 10.1111/j.1365-3156.2008.02036.x. 18318697 10.1111/j.1365-3156.2008.02036.x

[7] World Health Organization, Geneva. Mental disorders. WHO; 2024. https://www.who.int/news-room/fact-sheets/detail/mental-disorders.

[8] Huizink AC, Mulder EJ, Robles de Medina PG, Visser GH, Buitelaar JK. Is pregnancy anxiety a distinctive syndrome? Early Hum Dev. 2004;79(2):81–91. 10.1016/j.earlhumdev.2004.04.014. 15324989 10.1016/j.earlhumdev.2004.04.014

[9] Dunkel Schetter C, Tanner L. Anxiety, depression and stress in pregnancy: implications for mothers, children, research, and practice. Curr Opin Psychiatry. 2012;25(2):141–8. 10.1097/YCO.0b013e3283503680. 22262028 10.1097/YCO.0b013e3283503680 PMC4447112

[10] Lancaster CA, Gold KJ, Flynn HA, Yoo H, Marcus SM, Davis MM. Risk factors for depressive symptoms during pregnancy: a systematic review. Am J Obstet Gynecol. 2010;202(1):5–14. 10.1016/j.ajog.2009.09.007. 20096252 10.1016/j.ajog.2009.09.007 PMC2919747

[11] Fisher J, Cabral de Mello M, Patel V, Rahman A, Tran T, Holton S, et al. Prevalence and determinants of common perinatal mental disorders in women in low- and lower-middle-income countries: a systematic review. Bull World Health Organ. 2012;90(2):139–49. 10.2471/BLT.11.091850. 10.2471/BLT.11.091850 PMC3302553 22423165

[12] Bhushan NL, Krupp K, Jaykrishna P, Ravi K, Khan A, Shidhaye R, Kiplagat S, Srinivas V, Madhivanan P. The association between social support through contacts with Accredited Social Health Activists (ASHAs) and antenatal anxiety among women in Mysore, India: a cross-sectional study. Soc Psychiatry PsychiatrEpidemiol. 2020;55(10):1323–33. 10.1007/s00127-020-01864-5. 10.1007/s00127-020-01854-4 PMC7483323 32146484

[13] Huizink AC, Delforterie MJ, Scheinin NM, Tolvanen M, Karlsson L, Karlsson H. Adaption of pregnancy anxiety questionnaire-revised for all pregnant women regardless of parity: PRAQ-R2. Arch Womens Ment Health. 2016;19(1):125–32. 10.1007/s00737-015-0531-2. 25971851 10.1007/s00737-015-0531-2 PMC4728175

[14] Hamed Mohamed W, El nemer A. Pregnancy related to anxiety among chronically ill women. Mansoura Nurs J. 2018;5(3):147–57. 10.21608/mnj.2018.176492.

[15] Nath A, Venkatesh S, Balan S, Metgud CS, Krishna M, Murthy GVS. The prevalence and determinants of pregnancy-related anxiety amongst pregnant women at less than 24 weeks of pregnancy in Bangalore, Southern India. Int J Womens Health. 2019;11:241–8. 10.2147/IJWH.S193306. 31114392 10.2147/IJWH.S193306 PMC6489575

[16] Jyothi Kantipudi S, Kannan GK, Viswanathan S, Ranganathan S, Menon J, Ramanathan S. Antenatal depression and generalized anxiety disorder in a tertiary hospital in South India. Indian J Psychol Med. 2020;42(6):513–8. 10.1177/0253717620928440. 33354075 10.1177/0253717620928440 PMC7735237

[17] Naja S, Al Kubaisi N, Singh R, Bougmiza I. Generalized and pregnancy-related anxiety prevalence and predictors among pregnant women attending primary health care in Qatar, 2018–2019. Heliyon. 2020;6(10):e05264. 10.1016/j.heliyon.2020.e05264. 33134579 10.1016/j.heliyon.2020.e05264 PMC7586091

[18] Wall V, Premji SS, Letourneau N, McCaffrey G, Nyanza EC. Factors associated with pregnancy-related anxiety in Tanzanian women: a cross sectional study. BMJ Open. 2018;8(6):e020056. 10.1136/bmjopen-2017-020056. 29866722 10.1136/bmjopen-2017-020056 PMC5988139

[19] Tarafa H, Alemayehu Y, Nigussie M. Factors associated with pregnancy-related anxiety among pregnant women attending antenatal care follow-up at Bedelle general hospital and Metu Karl comprehensive specialized hospital, Southwest Ethiopia. Front Psychiatry. 2022;13:938277. 10.3389/fpsyt.2022.938277. 36213901 10.3389/fpsyt.2022.938277 PMC9537765

[20] Sahoo S, Gill G, Sikka P, Nehra R. Antenatal depression and anxiety in Indian women: A systematic review. Ind Psychiatry J. 2023;32(2):222–33. 10.4103/ipj.ipj_156_22. 10.4103/ipj.ipj_156_22 PMC10756614 38161466

[21] Bagade V, Mhatre B. Prevalence of pregnancy related anxiety in pregnant women in southern fringes of Pune, India. Int J Health Sci Res. 2021;11(10):41–9. 10.52403/ijhsr.20211007.

[22] Gourounti K, Anagnostopoulos F, Lykeridou K. Coping strategies as psychological risk factor for antenatal anxiety, worries, and depression among Greek women. Arch Womens Ment Health. 2013;16(5):353–61. 10.1007/s00737-013-0338-y. 23558945 10.1007/s00737-013-0338-y

[23] Dencker A, Nilsson C, Begley C, Jangsten E, Mollberg M, Patel H, Wigert H, Hessman E, Sjöblom H, Sparud-Lundin C. Causes and outcomes in studies of fear of childbirth: A systematic review. Women Birth. 2019;32(2):99–111. 10.1016/j.wombi.2018.07.004. 30115515 10.1016/j.wombi.2018.07.004

[24] Rosario MK, Premji SS, Nyanza EC, Bouchal SR, Este D. A qualitative study of pregnancy-related anxiety among women in Tanzania. BMJ Open. 2017;7(8):e016072. 10.1136/bmjopen-2017-016072. 28775187 10.1136/bmjopen-2017-016072 PMC5629724

[25] Neggers Y, Goldenberg R, Cliver S, Hauth J. The relationship between psychosocial profile, health practices, and pregnancy outcomes. Acta Obstet Gynecol Scand. 2006;85(3):277–85. 10.1080/00016340600566121. 16553174 10.1080/00016340600566121

[26] Lobel M, Cannella DL, Graham JE, DeVincent C, Schneider J, Meyer BA. Pregnancy-specific stress, prenatal health behaviors, and birth outcomes. Health Psychol. 2008;27(5):604–15. 10.1037/a0013242. 18823187 10.1037/a0013242

[27] Westerneng M, Witteveen AB, Warmelink JC, Spelten E, Honig A, de Cock P. Pregnancy-specific anxiety and its association with background characteristics and health-related behaviors in a low-risk population. Compr Psychiatry. 2017;75:6–13. 10.1016/j.comppsych.2017.02.002. 28279817 10.1016/j.comppsych.2017.02.002

[28] Alipour Z, Lamyian M, Hajizadeh E, Vafaei MA. The association between antenatal anxiety and fear of childbirth in nulliparous women: a prospective study. Iran J Nurs Midwifery Res. 2011 Spring;16(2):169–73. PMC3249768 22224102

[29] Van Heyningen T, Honikman S, Myer L, Onah MN, Field S, Tomlinson M. Prevalence and predictors of anxiety disorders amongst low-income pregnant women in urban South Africa: a cross-sectional study. Arch Womens Ment Health. 2017;20(6):765–75. 10.1007/s00737-017-0768-z 28852868 10.1007/s00737-017-0768-z PMC6086488

[30] Elgzar WT, Alshahrani MS, Ibrahim HA. Mode of delivery preferences: the role of childbirth fear among nulliparous women. Front Psychol. 2023;14:1221133. 10.3389/fpsyg.2023.1221133. 38034315 10.3389/fpsyg.2023.1221133 PMC10687373

[31] Mohamamdirizi S, Mohamadirizi M, Mohamadirizi S. The comparison of fear of childbirth and sense of coherence among low-risk and high-risk pregnancy women. J Educ Health Promot. 2018;7:143. 10.4103/jehp.jehp_179_17. 30596115 10.4103/jehp.jehp_179_17 PMC6282476

[32] Kristensen IH, Simonsen M, Trillingsgaard T, Pontoppidan M, Kronborg H. First-time mothers’ confidence mood and stress in the first months postpartum. A cohort study. Sex ReprodHealthc. 2018;17:43–9. 10.1016/j.srhc.2018.06.003. 10.1016/j.srhc.2018.06.003 30193719

[33] Serçekuş P, Okumuş H. Fears associated with childbirth among nulliparous women in Turkey. Midwifery. 2009;25(2):155–. 10.1016/j.midw.2007.02.005. 62. 17600599 10.1016/j.midw.2007.02.005

[34] Mainali A, Infanti JJ, Thapa SB, Jacobsen GW, Larose TL. Anxiety and depression in pregnant women who have experienced a previous perinatal loss: a case-cohort study from Scandinavia. BMC Pregnancy Childbirth. 2023;23(1):111. 10.1186/s12884-022-05318-2. 36782148 10.1186/s12884-022-05318-2 PMC9923894

[35] Omar N, Major S, Mohsen M, Al Tamimi H, El Taher F, Kilshaw S. Culpability, blame, and stigma after pregnancy loss in Qatar. BMC Pregnancy Childbirth. 2019;19(1):215. 10.1186/s12884-019-2354-z. 31242874 10.1186/s12884-019-2354-z PMC6595691

[36] Thapar AK, Thapar A. Psychological sequelae of miscarriage: a controlled study using the general health questionnaire and the hospital anxiety and depression scale. Br J Gen Pract. 1992;42(356):94–6. 1493042 PMC1371991

[37] Chojenta C, Harris S, Reilly N, Forder P, Austin MP, Loxton D. History of pregnancy loss increases the risk of mental health problems in subsequent pregnancies but not in the postpartum. PLoS ONE. 2014;9(4):e95038. 10.1371/journal.pone.0095038. 24733508 10.1371/journal.pone.0095038 PMC3986356

[38] Choudhary N, Sangwan RK, Kumar M, Khetan M, Huda RK, Arora S, Gill R, Valecha V, Babu BV. Anxiety among pregnant women in the second and third trimesters: A qualitative study. J Affect Disorders Rep. 24;101045. 10.1016/j.jadr.2026.101045.

[39] Sangwan RK, Kumar D. Health and Society*.* New Delhi: Academic Publication; 2019. ISBN: 978-93-83931-65-1.

